# The Rapidity of the Circulation of the Blood

**Published:** 1885-10

**Authors:** 


### The Rapidity of the Circulation of the Blood.

Dr. Robert Meade Smith, professor of comparative physiology in the University of Pennsylvania, recently read a paper before the College of Physicians of Philadelphia, which was subsequently published in the Medical and Surgical Reporter, in its issues of May 31st and June 7th, in which he presents a report of some interesting experiments conducted by himself with a view to ascertainining the time required by the blood for making one complete circuit of the body.

His conclusions are somewhat at variance with the generally accepted opinions on this subject, and are worthy of careful consideration. The paper opens with a description and criticism of the method, that of Hering, which has heretofore been employed in determining this question. It consists in injecting into one of the jugular veins, towards the heart, a solution of some salt, whose presence in the blood can be readily recognized by chemical tests, and discovering how soon after its injection it appears in the blood coming from the corresponding vein on the opposite side of the neck. The accuracy of this method depends upon the correctness of two assumptions, namely, that the salt used has been carried mechanically by the blood, without diffusing through it, with the same velocity as the blood itself; and, secondly, that the velocity thus determined by the infusion method, is the mean velocity of the blood mass. Dr. Smith demonstrates that both of these assumptions are unwarrantable. He shows that the element of diffusion forms a considerable item in the rapidity with which the solution of salt completes the circuit of the body. In his method, which consists in the injection of defibrinated pigeon's blood, this source of error does not exist. The red blood corpuscles of the pigeon have a short diameter, smaller than the diameter of mammalian blood discs, and are thus not interfered with in their passage through the circulation of a mammal. Dr. Smith's apparatus is of a very ingenious and delicate order. His kymographion and electro-magnetic lever are so adjusted as to accurately record the pulsations of the animal being experimented upon. A collection of twenty-four watch-crystals is attached to a horizontal glass disc, which is uniformly rotated by clock-work at any desired speed. Into the watch-crystals upon this disc, so rotating, flows the blood from the opposite jugular vein to that into which the pigeon's blood was injected, and the exact time of the discharge into each crystal noted. The examination of the blood as it is discharged into the watch-crystals reveals the appearance of the corpuscles of the pigeon's blood, and the time intervening between their injection into one jugular vein and the exit from the other being accurately determined by the kymographion, the time consumed by their circuit of the body is thus discovered.

In the first experiment, on a dog weighing 18 kilogrammes (about 40 lbs.), the time required by the pigeon corpuscles to pass from the left to the right jugular was 20 seconds. In the second experiment, the dog weighing 10 kilogrammes (about 22 lbs.), the circuit was made in 17 seconds, and the average time consumed in 10 experiments on dogs was 17½ seconds, as against 15 22-100 reported by Vicrordt, as required by potassium ferrocyanide to make the circuit of the circulation in dogs of corresponding average weight.

In rabbits, the average time intervening between the injection of pigeon's blood and the appearrance of the corpuscles, was 11 seconds, as against 6 9-10 seconds consumed in the passage of the potassium salt. We have here, then, a difference of nearly 15 per cent. in the case of the dog, and over 59 per cent. in the case of the rabbit, which must be accounted for through the diffusion of the potassium salt. The rapidity of circulation is greater in small animals than in large, not only on account of the shorter path to be traversed, as insisted upon by Vicrordt, but because smaller animals contain, both absolutely and relatively, as to body weight, a smaller volume of blood, and since it has been shown that diffusion has something to do with the result, the salt will diffuse more rapidly through a small volume of fluid than a large one. This fact accounts for the difference in the diffusion as noted in the rabbit and the dog.

Blood moves more rapidly in the center of the blood vessel than at the sides, and the figures as noted above, must, therefore, be corrected so as to have them represent the mean circulation. Dr. Smith conducted another series of experiments with carmine, in order to determine the difference in the rapidity of the flow at the margin and in the center of the blood current. As a result of these experiments, he places the average time to complete the circulation in a dog weighing 10 kilogrammes (about 22 lbs.) at between 25 and 30 seconds, as against 17½ seconds, the average time consumed between the injection of the pigeon's blood and the appearance of pigeon's corpuscles in the opposite jugular vein.

The paper then goes on to discuss the statement madeby Vicrordt, that moderate irritation of the vagi lengthens the time of circulation, by reducing the number of pulsations of the heart. Dr. Smith concludes from his experiments that while irritation of the vagus diminishes the frequency of the cardiac contractions, the contractions are more forcible, each one throwing out a sufficiently larger volume of blood to compensate for the diminished frequency. Irritation of the vagi does not, therefore, increase the length of time required to complete the circulation of the whole volume of blood.

